# Investigating the association of ventral and dorsal striatal dysfunction during reward anticipation with negative symptoms in patients with schizophrenia and healthy individuals

**DOI:** 10.1371/journal.pone.0198215

**Published:** 2018-06-18

**Authors:** Marta Stepien, Andrei Manoliu, Roman Kubli, Karoline Schneider, Philippe N. Tobler, Erich Seifritz, Marcus Herdener, Stefan Kaiser, Matthias Kirschner

**Affiliations:** 1 Department of Psychiatry, Psychotherapy and Psychosomatics, Psychiatric Hospital, University of Zurich, Zurich, Switzerland; 2 Laboratory for Social and Neural Systems Research, Department of Economics, University of Zurich, Zurich, Switzerland; 3 Neuroscience Center Zurich, University of Zurich, Zurich, Switzerland; 4 Zurich Center for Integrative Human Physiology, University of Zurich, Zurich, Switzerland; 5 Center for Addictive Disorders, Psychiatric Hospital, University of Zurich, Zurich, Switzerland; 6 Division of Adult Psychiatry, Department of Mental Health and Psychiatry, Geneva University Hospitals, Chemin du Petit-Bel-Air, Chêne-Bourg, Switzerland; Maastricht University, NETHERLANDS

## Abstract

**Background:**

Negative symptoms are a core feature of schizophrenia and also found in healthy individuals in subclinical forms. According to the current literature the two negative symptom domains, apathy and diminished expression may have different underlying neural mechanisms. Previous observations suggest that striatal dysfunction is associated with apathy in schizophrenia. However, it is unclear whether apathy is specifically related to ventral or dorsal striatal alterations. Here, we investigated striatal dysfunction during reward anticipation in patients with schizophrenia and a non-clinical population, to determine whether it is associated with apathy.

**Methods:**

Chronic schizophrenia patients (n = 16) and healthy controls (n = 23) underwent an event- related functional MRI, while performing a variant of the Monetary Incentive Delay Task. The two negative symptom domains were assessed in both groups using the Brief Negative Symptoms Scale.

**Results:**

In schizophrenia patients, we saw a strong negative correlation between apathy and ventral and dorsal striatal activation during reward anticipation. In contrast, there was no correlation with diminished expression. In healthy controls, apathy was not correlated with ventral or dorsal striatal activation during reward anticipation.

**Conclusion:**

This study replicates our previous findings of a correlation between ventral striatal activity and apathy but not diminished expression in chronic schizophrenia patients. The association between apathy and reduced dorsal striatal activity during reward anticipation suggests that impaired action-outcome selection is involved in the pathophysiology of motivational deficits in schizophrenia.

## Introduction

Negative symptoms (NS) are an integral feature of schizophrenia [1]. According to the current consensus, they can be separated into two domains: the apathy domain, consisting of avolition, anhedonia, and asociality and the diminished expression domain, consisting of blunted affect and alogia [2–4]. Moreover, recent studies have shown that this distinction into two subgroups is clearly observable in patients, showing clinically meaningful differences in symptom presentation [5]. Accumulating evidence suggests that both factors are caused by a different neurobiological and behavioral mechanisms [6–8]. However, due to its association with poorer social functioning and strong impact on recovery, understanding the pathophysiology of apathy is highly relevant [5,9].

Apathy can be defined as a quantitative reduction in goal-directed behavior due to diminished motivation [10–13]. According to the extensive evidence coming from animal and human research, dopamine (DA) mediates anticipation of rewards, as well as motivation to obtain them, and is therefore considered essential for goal-directed behavior [14,15]. In particular, mesolimbic dopaminergic projections from the ventral tegmental area (VTA) to the ventral striatum (VS) are thought to have a critical role in reward processing, especially reward “wanting” that generates motivation [8,15,16]. In addition, nigrostriatal dopaminergic projections from the substantia nigra to the dorsal part of the striatum are implicated in action outcome selection and are closely related to goal-directed behavior [15,17]. Current literature provides compelling evidence, that dysfunctions in reward processing play a crucial role for global negative symptoms, which are predominantly characterized by their association with ventral striatal hypoactivation [18–20]. Nevertheless, the picture of biological and behavioral underpinnings of apathy is just starting to emerge [14,18]. Even though some researchers have already focused on disentangling the two negative symptom dimensions on a behavioral and neural level, data are still very limited [6,21,22]. So far, most studies suggest an association between apathy and reduced ventral striatal activation during reward anticipation [6,8,23,24]. In contrast, recent research proposed that dysfunction of the dorsal striatum (DS) during reward anticipation might additionally be involved in the pathophysiology of apathy [25–28]. Indeed, several neuroimaging studies have investigated the role of ventral and dorsal striatal dysfunction in motivational deficits during reinforcement learning [29], gain and loss avoidance [30] or resting state functional connectivity [28]. However, only one neuroimaging study has directly compared striatal activation during reward anticipation with measures of apathy and diminished expression [6] and only one discussed ventral and dorsal striatal dysfunction during reward anticipation in the expression of avolition [26]. Therefore, more research is needed to elucidate the specific role of ventral and dorsal striatal activity in the complex pathophysiology of apathy.

While most studies have focused on the neural underpinnings of negative symptoms in schizophrenia, recent findings suggest that negative symptoms are also common in subclinical form in the general population [31–33]. Noteworthy, the presence of negative symptoms was particularly distressful for the affected individuals [31] and associated with an increased risk of developing psychiatric disorders [32]. On a neural level, data from studies on unaffected first- degree relatives of schizophrenia patients indicate the presence of striatal dysfunction during expectation of reward [34–38]. In addition, ventral striatal impairments have been linked to schizotypal personality traits, trait-anhedonia or psychotic-like-symptoms in the general population [39–43]. However, the evidence from healthy individuals without a genetic risk of developing psychiatric disorders is scarce and inconclusive. Therefore, it is critical to elucidate the dimensional aspects of apathy and striatal dysfunction.

The aim of the present study was threefold. First, we sought to replicate our previous findings of ventral striatal dysfunction in patients with schizophrenia as specific neural substrate for apathy in an independent sample. Second, we investigated the role of the DS in the pathophysiology of apathy. Third, we tested whether the association between negative symptoms and striatal dysfunction could also be observed in a non-clinical population. We hypothesized that more severe apathy, but not diminished expression, is associated with ventral and dorsal striatal hypoactivation during the expectation of reward in healthy participants as well as in individuals with schizophrenia.

## Methods and materials

### Participants

Patients with schizophrenia (n = 18) were recruited from inpatient (n = 14) and outpatient (n = 4) units of the University Psychiatric Hospital in Zurich and from affiliated institutions. All inpatients included in the present study were at the end of their hospitalization, participated in a multimodal treatment program and engaged in activities outside the hospital, allowing us to assess negative symptoms. Please note that in Switzerland, the average duration of inpatient treatment of patients with schizophrenia is approximately 40 days [44], which means that most of our inpatients would have been treated as outpatients in other health care systems. Outpatients were in protected working/educational programs (n = 4). Healthy controls (HC) (n = 26) were recruited from the general community. Diagnosis of schizophrenia or schizoaffective disorder were confirmed with the structured Mini International Neuropsychiatric Interview for DSM IV (MINI). Individuals with any other Axis I DSM IV disorder, in particular major depression or current substance use disorder, were excluded from the study. All patients with schizophrenia were clinically stable and received a stable dose of medication for at least two weeks. The study was approved by the local ethics committee of the Canton Zurich. All participants signed the written informed consent in accordance with the Declaration of Helsinki. The capability of SZ patients to give informed consent was evaluated by the treating psychiatrist.

### Clinical and neuropsychological assessment

The Brief Negative Symptoms Scale (BNSS) [45] was administered to all participants in order to assess the severity of negative symptoms in two dimensions: apathy and diminished expression. The apathy dimension score (deficits in motivation and pleasure) consisted of avolition (items: 7, 8), asociality (items: 5,6) and anhedonia (items: 1, 2, 3), while the diminished expression score, included blunted affect (items: 9, 10, 11) and alogia (items: 12, 13).

To identify depressive symptoms, we used the Calgary Depression Scale for Schizophrenia (CDSS) [46] in patients and Beck’s Depression Inventory (BDI) in HC [47].

### Experimental design and task

**Fig 1:**
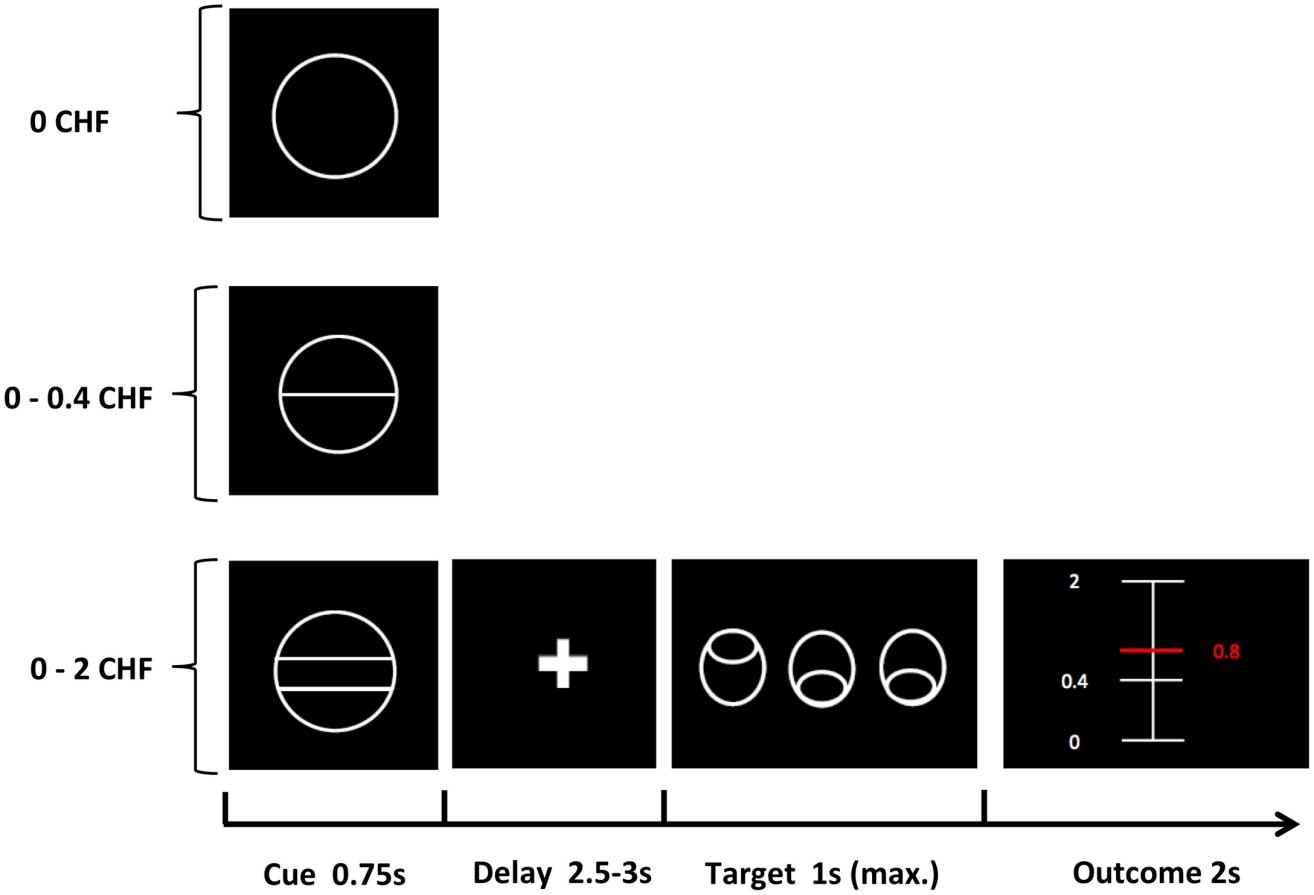
Schematic illustration of the variant of Monetary Incentive Delay Task (MID) (adapted from Kirschner et al. [6]). In each trial, participants saw 1 of 3 cues, indicating the amount of money to be won. After a delay period, participants had to identify an outlier from an array of 3 circles by pressing a correct button (either left or right). Immediately after a correct button press, participants were informed via visual feedback about the amount of money they had won during the current trial. A red horizontal line on the column ranging from the minimal amount (0 CHF) to the maximal win amount (2.0 CHF) indicated the precise amount of money won in each trial.

We employed a modified version of the Monetary Incentive Delay Task (MID) [48], which was originally developed by Simon et al. [43] and used in our previous studies [6,49,50] (Fig 1). In this variant, reward amount was directly determined by the behavior of the participants (response time) (Fig A in S1 File). This adaptation allowed us to investigate the motivational properties of reward anticipation in the presence of strong action-outcome contingencies. Participants were informed that the complete amount of money, accumulated during the 2 experimental blocks, will be paid out to them at the end of the experiment. At the beginning of each trial, 1 of 3 cues was presented (cue duration 0.75s), indicating the maximum possible monetary amount to be won (2 CHF, 0.4 CHF, 0 CHF). After a delay period (2.5- 3s), participants had to identify an outlier (appearing on the left or right) from an array of 3 circles, by pressing a button (right or left; middle stimulus was never an outlier) as fast as possible. If they pressed the correct button, participants immediately received feedback informing them about the amount of money they had won in the current trial (feedback duration: 2s) (Fig 1). Error trials were defined as wrong or late response (after 1s). The actual amount of money to be won for each trial was calculated based on the response times from the previous 15 individual trials [6,43]. This approach was used to account for individual differences in response time and assure high rewards in both groups. Participants performed 2 training sessions (36 trials per session), 1 outside and 1 inside the scanner. The experiment consisted of 2 runs, with each run consisting of 36 trials of about 10s each. The intertrial interval was jittered from 1 to 9s with a mean of 3.5s. Each run lasted about 6 min. The task was implemented using the MATLAB toolboxes Cogent 2000 and Cogent Graphics.

### Image acquisition

Functional and structural images were obtained at the MR Centre of the Psychiatric Hospital, University of Zurich, using a Philips Achieva 3.0 T scanner with a 32-channel SENSE head coil (Philips, Best, The Netherlands). Functional scans were collected in 2 runs with 195 images in each run, using T2- weighted echo-planar image (EPI) sequence over a whole brain with 38 slices acquired in ascending order. Acquired in-plane resolution was 3×3mm^2^, 3mm slice thickness and 0.5mm gap width over a field of view of 240×240mm^2^, a repetition/echo time (TR/TE) of 2000/25ms and a flip angle of 82°. To eliminate the influence of T1 saturation effects, the first five scans were discarded. An ultrafast gradient echo T1- weighted sequence in 160 sagittal plane slices of 240 x 240 mm with voxel size 1 x 1 x1 mm was acquired for anatomic reference.

### Data analysis

Statistical analysis of demographic, neuropsychological and behavioral data, as well as correlations with symptom dimensions were performed using SPSS (IBM SPSS Statistics, Version 23.0., Armonk, NY: IBM Corp). Imaging data were analyzed in SPM 8 (Statistical Parametric Mapping, Wellcome Trust Centre for Neuroimaging, London, UK).

### Behavioral data analysis

Response time (RT), defined as time between target presentation and pressing the correct answer button was the main behavioral outcome measure. We performed a two- way repeated measures ANOVA with group affiliation (SZ, HC) as between-subject factor and reward condition (neutral, low, high) as within-subject factor. To investigate potential differences between groups, we used two-tailed independent sample t-tests. To interrogate non-normally distributed data (as assessed with Kolmogorov-Smirnov test) Mann-Whitney U-tests were applied.

### Image pre-processing

All preprocessing steps were performed using SPM8. Functional images were corrected for differences in the time of slice acquisition in ascending order with reference to the 19^th^ slice. To correct for head motion, we used the Realign and Unwarp function of SPM 8 and allowed translational head motion limited to ± 3mm. Therefore, to assure adequate quality of fMRI data all participants with translational head movement ≥ 3mm or extensive signal dropout in subcortical and prefrontal regions in the EPI sequences were excluded. Next the data underwent segmentation, bias correction and spatial normalization. Finally, spatial smoothing was performed, using a 6 mm full- width at half maximal Gaussian kernel.

### First and second- level analysis

At the first level, we constructed a general linear model (GLM), consisting of 12 regressors. Three separate regressors (duration 3.25–3.75s) were modelled for the reward anticipation phase: anticipation of no reward (0 CHF), low reward (0–0.4 CHF) and high reward (0–2.0 CHF). To represent the outcome phases, we included 1 regressor for each reward condition (3 basic regressors). Moreover, 2 parametrically modulated regressors were modelled, corresponding to the actual outcome amount of each trial (1 for the low reward, 1 for the high reward). Target presentation (1 regressor) and error trials (3 regressors) were included as additional regressors of no interest. Please note that we used the unwarp method of spm8 during preprocessing to correct for movement artefacts and therefore do not include motion parameters in the first level fMRI model following the procedure described in the spm8 manual (Chapter 3 Realign and Unwarp). For more detail about the underlying theory: Andersson et al. [51]. All explanatory variables were convolved with the canonical hemodynamic response function. For the analysis of the reward anticipation phase we calculated the main contrast of interest: anticipation of high reward (2 CHF) versus anticipation of no reward (0 CHF). The individual contrast images of all participants were included in the random- effects model, at the second level of analysis. Within- group activation was assessed using 1 sample t-tests and between- group activation differences using 2-sample t-tests.

### Region of interest image analysis

Based on our a priori hypothesis, we defined the VS and the DS as regions of interest (ROIs). Masks for the VS and DS ROIs were taken from the study of Mawlawi and colleagues [52], which defined the striatal subregions based on the atlas of Mai and colleagues [53]. The VS mask includes the nucleus accumbens, ventral caudate, and ventral putamen, while the DS includes dorsal caudate and dorsal putamen. For all analyses, we used one VS ROI (including left and right VS) and one DS ROI (including left and right DS). The ROI analyses were performed at the voxel level with family-wise error (FWE) correction for multiple comparisons in each ROI (*p* = 0.05). Mean percent signal change was extracted from the ROIs using the REX toolbox for SPM 8. ROIs and task activation were visualized using sweetView (http://www.sweetneuron.at/wp/sweetview/).

### Correlation analyses

We tested the main hypotheses by calculating bivariate Spearman correlations (rho) between the negative symptoms dimensions apathy and diminished expression, and percent signal change in the dorsal and ventral striatum. To test for potential differences between these correlations, we performed the Steiger test for dependent correlation coefficients. Additionally, we performed correlation analyses with potential confounding variables and striatal activation during reward anticipation.

## Results

### Sample characteristic

The initial sample of the study comprised 44 participants (SZ = 18, HC = 26). From the 18 recruited patients, 2 were excluded, as they did not complete the whole experimental procedure. 2 HC were excluded due to signal dropout in functional images and 1 because of excessive movements in the scanner (≥ 3mm). The final sample consisted of 39 subjects: 16 SZ and 23 HC. Hence, 11% of participants from the SZ group and 9% from the HC group were excluded. The final sample consisted of 39 subjects: 16 SZ and 23 HC Participant characteristics, clinical data and group comparisons are summarized in Table 1.

**Table 1:** Clinical and demographic characteristics.

Characteristics	SZ (n = 16)	HC (n = 23)	Statistical test	p value
Age	32.6 (9.2)	29.5 (6.6)	U = 231.5	0.177
Sex	2w,14m	12w,11m	U = 257.0	0.037
Education, yr.	12.0 (1.8)	15.2 (2.1)	t = 5.0	0.00001
DUP, days	619.7 (833.1)			
Chlorpromazine equivalents, mg/d	482.3 (579.3)			
**BNSS score**				
Apathy	15.6 (9.4)	2.0 (2.8)	t = -5.6	0.00003
Diminished Expression	8.5 (8.5)	1.0 (2.0)	t = -3.5	0.00003
Total	25.1 (13.9)	3.0 (3.4)	t = -6.2	0.00001
**PANSS score**				
Positive	7.2 (2.5)			
Negative	15.2 (7.2)			
Disorganized	5.7 (2.1)			
Excited	4.8 (1.2)			
Depressed	6.3 (1.9)			
Total	54.3 (11.6)			
CDSS	2.8 (2.1)			
BDI	9.1 (8.4)	3.2 (3.7)	-2.6	0.005

Notes: Data are presented as means and standard deviations. BDI = Beck Depression Inventory; BNSS = Brief Negative Symptom Scale; CDSS = Calgary Depression Scale for Schizophrenia; DUP = Duration of untreated psychosis; PANSS = Positive and Negative Syndrome Scale

### Behavioral data

We log-transformed the raw RT data to account for non-normality and inhomogeneity of variance. The repeated-measures ANOVA with reward condition as within-subject factor and group affiliation as between-subject factor revealed a significant main effect of reward (F(2,74) = 59.5, p <.001), reflecting faster responding on reward versus neutral trials and intact reward reward-related speeding. Post-hoc tests for each group separately confirmed that both groups were faster in the low reward trials versus neutral trials (HC: t(22) = 7.55, p <0.001; SZ: t(15) = 3.45, p = 0.004) and in high reward trials versus neutral trials (HC: t(22) = -10.20, p <0.001; SZ: t(15) = -3.45, p = 0.004). The main effect of group was also significant (F(1,37) = 12.1, p = .001), showing that SZ patients were generally slower than HC. Post-hoc pairwise comparison showed that this effect was significant in all three reward conditions (all p≤.001). Moreover, there was a significant reward*group interaction (F(2,74) = 14.5, p <.001), indicating smaller RT differences on incentive versus neutral trials in schizophrenia patients. In other words, reward lead to less speeding of responses in patients with SZ. While we did not find a significant correlation between the mean RT and one of the two negative symptom factors (apathy: r = .36, p = .17; diminished expression: r = .30, p = .25), global negative symptoms (BNSS total score) were significantly correlated with mean RT (r = .58, p = .02) in the schizophrenia group, but not in HC (r = -.24, p = .27). These findings suggest that besides categorical differences between HC and patients with SZ, individual levels of negative symptoms were associated with behavioral performance during the task. Group comparisons of total error rates and total gain are summarized in Table 2 and (Table A in S1 File).

**Table 2:** Behavioral results of the variant of the Monetary Incentive Delay Task.

	SZ (n = 16)	HC (n = 23)	Statistical test	p value
**Response time, ms**				
No reward	576.8 (96.0)	520.8 (56.5)	U = 259.0	0.03
No reward (log)	2.76 (0.07)	2.71 (0.05)	t = -2.1	0.04
Low reward	555.6 (102.8)	465.8 (48.6)	U = 285.0	0.003
Low reward (log)	2.74 (0.08)	2.67 (0.05)	t = -3.4	0.002
High reward	547.4 (99.0)	440.0 (400.6)	U = 324.0	≤0.001
High reward (log)	2.73 (0.08)	2.64 (0.04)	t = -4.6	≤0.001
Error rate	6.1 (5.6)	3.8 (2.5)	U = 234.0	0.159
Total gain, CHF	36.7 (3.6)	41.2 (4.2)	t = 3.1	0.003

Notes: Data are presented as means and standard deviations. log = log-transformed response time values. Potential group differences were investigated using 2-sample t-tests for continuous data. For non-normally distributed data we used Mann-Whitney U tests.

Given the group differences in the total amount of money won, we performed additional correlation analyses to investigate potential effects of striatal activation during reward anticipation and levels of negative symptoms on received monetary reward. We did not find any significant relationship between the total amount won and striatal activation during reward anticipation (SZ: VS: r_s_ = .30, p = .26; DS: r_s_ = .33, p = .22; HC: VS: r_s_ = -.12, p = .6; DS r_s_ = -.23, p = .29) or the total amount won and the apathy nor the diminished expression scores (SZ: apathy: r_s_ = .36, p = .17; diminished expression: r_s_ = .07, p = 0.80; HC: apathy: r_s_ = -.24, p = .27; diminished expression: r_s_ = -.30, p = .17). These additional analyses confirm that neither striatal activation during reward anticipation nor symptom levels of apathy were associated with the individual amount of money won during the experiment.

### Functional imaging data

**Fig 2:**
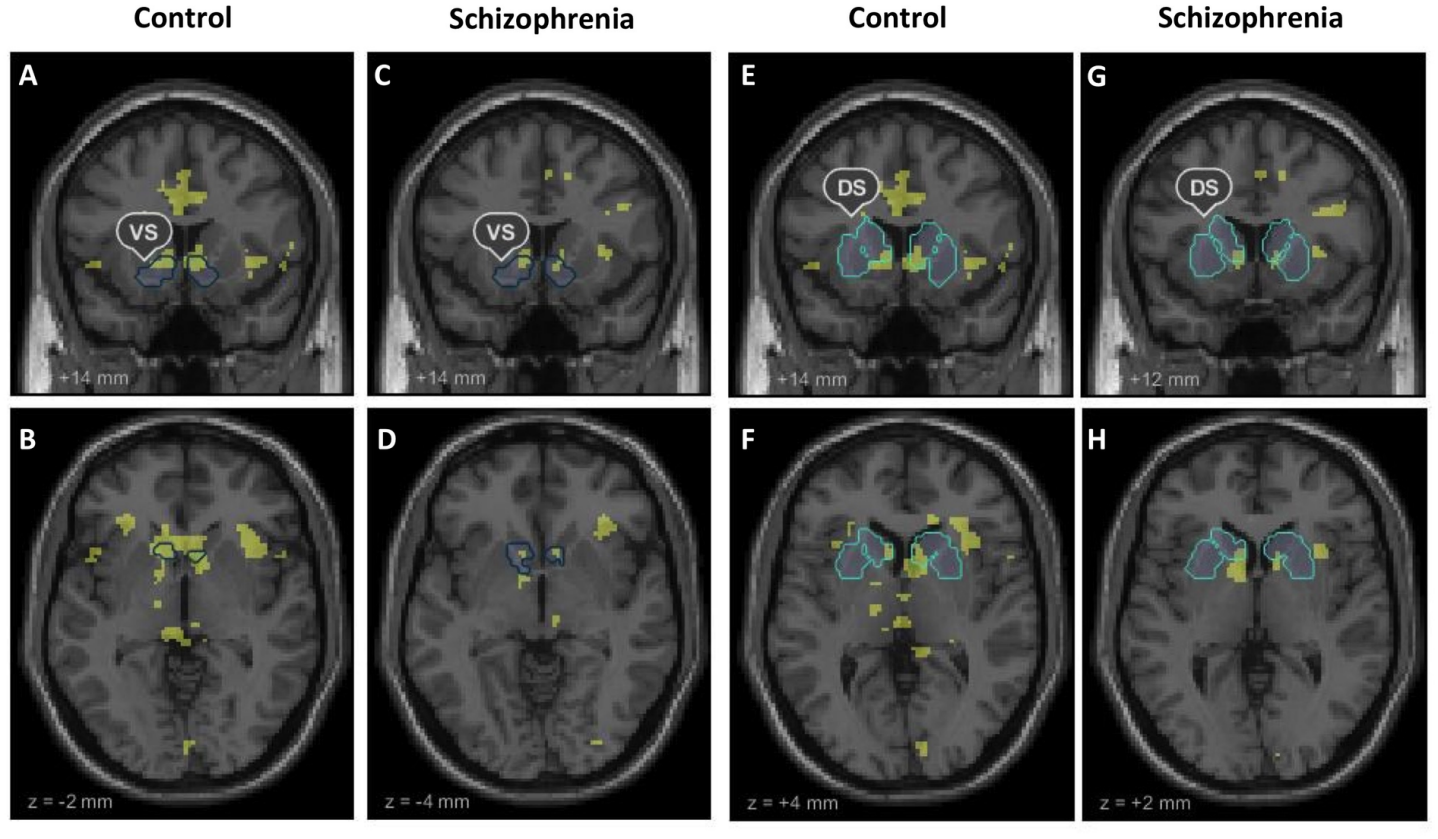
Whole brain within-group activation during reward anticipation. Whole brain within-group activation maps of the contrast anticipation of high reward versus no reward (p < 0.001, uncorrected). The within group t-maps are shown together with outlines of the ventral striatum (VS) and dorsal striatum (DS) our predefined regions of interest. (A), (E) Coronal and (B), (F) axial contrast images of healthy controls. (C), (G) Coronal and (D), (H) axial contrast images of patients with schizophrenia.

Our fMRI analysis focused on neural activation during anticipation of high reward versus anticipation of no reward in our predefined ROIs of the VS and DS. Ventral striatal signal in healthy controls and patients with schizophrenia was significantly stronger during the anticipation of high reward than anticipation of no reward (HC, left: cluster size = 218, t = 6.98; right: cluster size = 254, t = 8.59, both p<0.05, FWE-corrected; SZ, left: cluster size = 45, t = 5.68; right: cluster size = 44, t = 5.88, both p<0.05, FWE-corrected). Likewise, the dorsal striatum showed significantly stronger activation in both groups during high reward anticipation versus no reward anticipation (HC, left: cluster size = 69, t = 6.68; right: cluster size = 139, t = 9.64; SZ, left: cluster size = 17, t = 5.49; right: cluster size = 4, t = 5.11, all p <.05, FWE-corrected). Group comparisons of percent signal change during high reward anticipation versus no reward anticipation revealed no significant differences between HC and SZ, neither in the VS (t = .21, p = .84) nor in the DS (t = -.33, p = .74). Within-group activation during reward anticipation in the VS and DS are displayed in Fig 2 and (Table B in S1 File). Moreover, an explorative whole brain group comparison did not reveal any significant differences (at a whole-brain FWE (p <.05) cluster-level corrected threshold with cluster-inducing voxel-level threshold of p<0.001) in brain activations outside the ROIs.

### Correlation between anticipation of reward and negative symptoms dimensions

#### Ventral striatum

**Fig 3:**
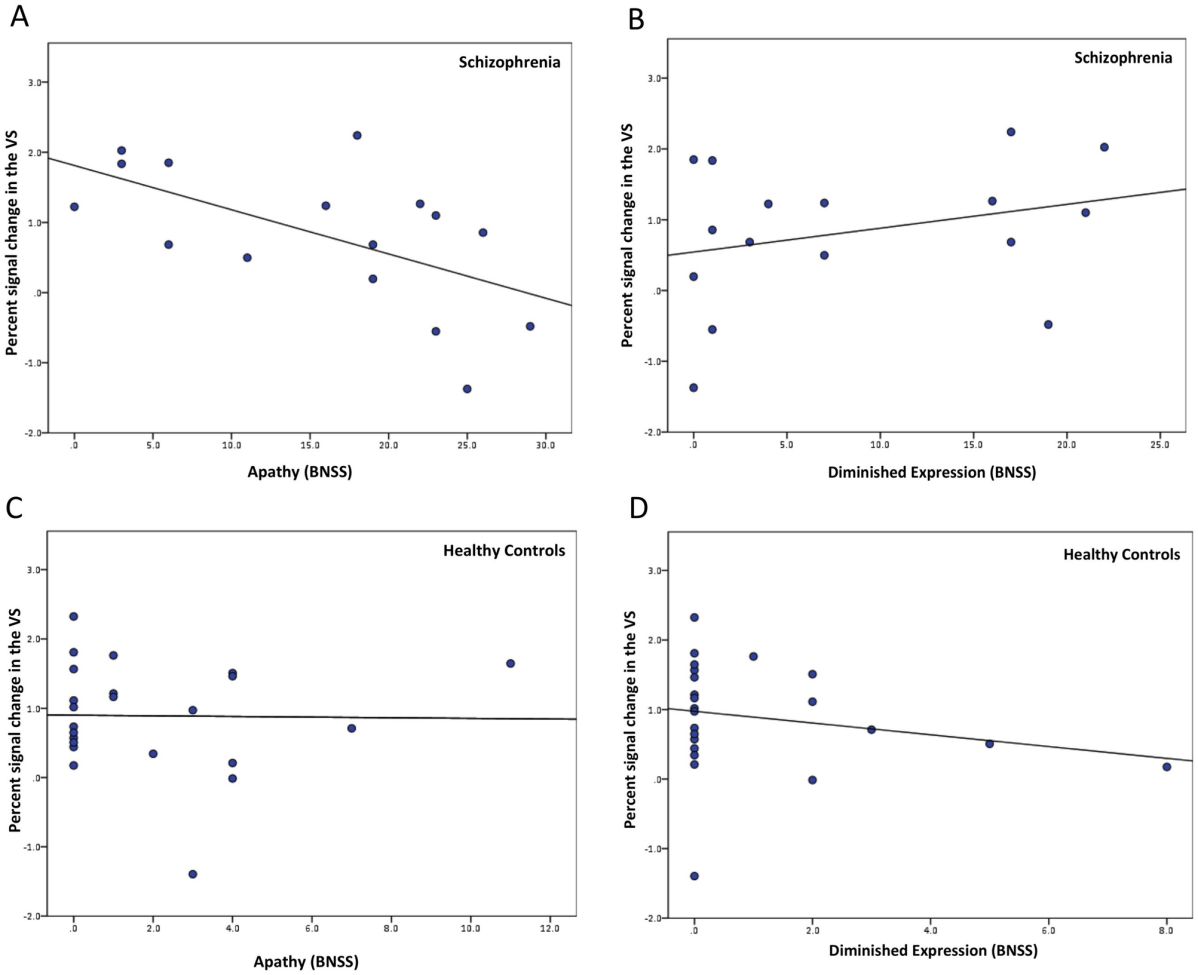
Correlations of the two negative symptom dimensions with VS activation during reward anticipation. Bivariate Spearman correlation of (A) apathy (r_s_ = -.59, p = .02) and (B) diminished expression (*r*_*s*_ = .30 p = .26) with percent signal change in the ventral striatum in patients with schizophrenia. Bivariate Spearman correlation of (C) apathy (r_s_ = -.06, p = .79) and (D) diminished expression (r_s_ = -.20, p = .35) with percent signal change in the ventral striatum in healthy controls. BNSS = Brief Negative Symptom Scale.

In line with our previous findings [6], the percent signal change in the ventral striatum during reward anticipation was significantly and negatively correlated with apathy (r_s_ = -.59, p = .02 in patients with schizophrenia (Fig 3A). Hence, individuals with more severe apathy showed less activation in ventral striatum during reward anticipation. As hypothesized, no significant correlation was found between the ventral striatal activation during reward anticipation and diminished expression (r_s_ = .30, p = .26, Fig 3B). These correlation coefficients differed significantly (z = -2.53, p = .01). Thus, hypoactivation of the ventral striatum was more strongly related to the apathy factor than to the diminished expression factor. In HC, we found no significant association between negative symptom dimensions and percent signal change during reward anticipation (apathy: r_s_ = -.06, p = .79; diminished expression: r_s_ = -.20, p = .35, Fig 3C and 3D). Also, when combining both factors in the BNSS total score, we found that this measure of global negative symptoms was not associated with VS activity in healthy controls (r_s_ = -.24, p = .27) (Fig B in S1 File).

#### Dorsal striatum

**Fig 4:**
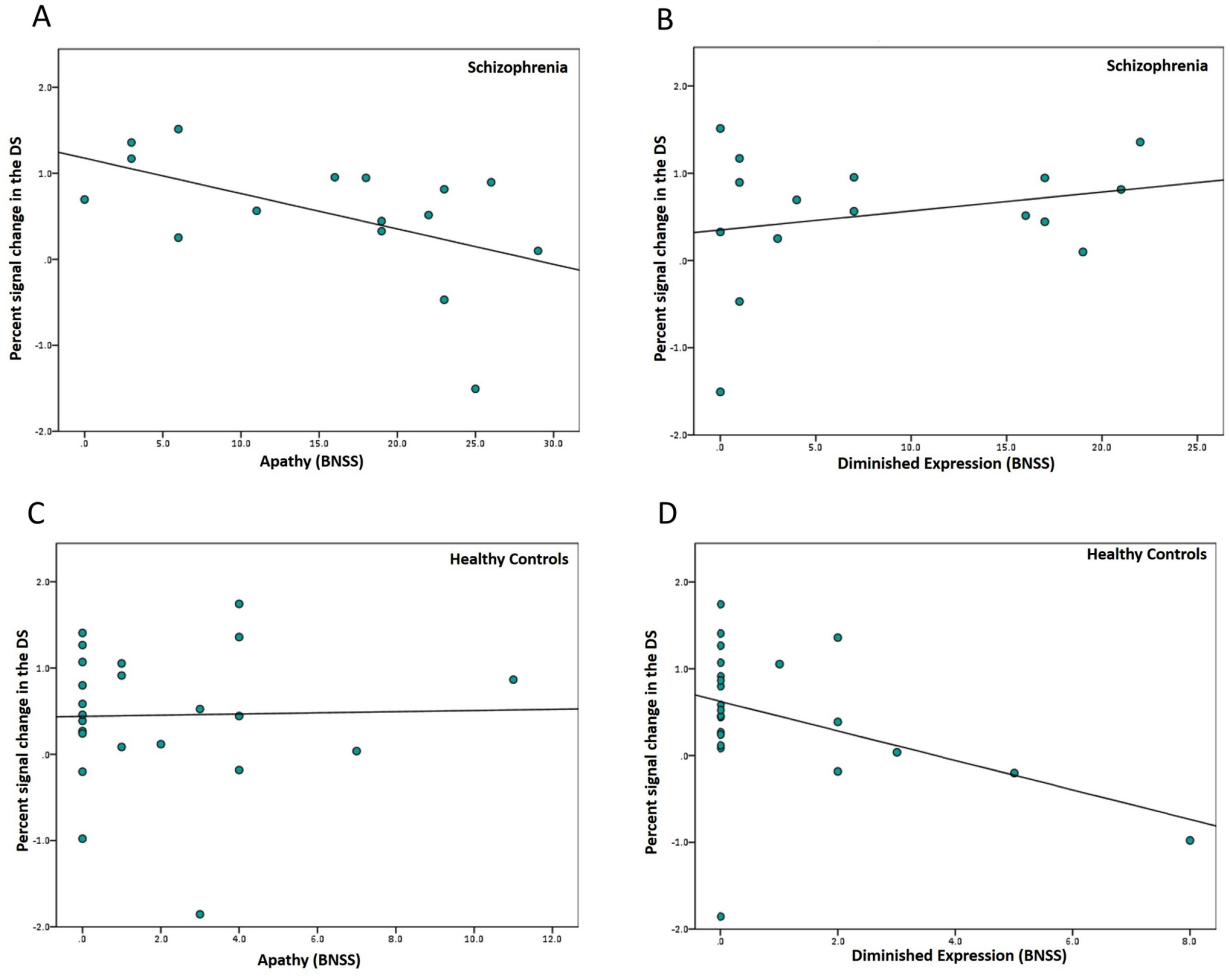
Correlations of the two negative symptom dimensions with DS activation during reward anticipation. Bivariate Spearman correlation of (A) apathy (*r*_*s*_ = -.56, p = .02) and (B) diminished expression (*r*_*s*_ = .30, p = .26) with percent signal change in the dorsal striatum in patients with schizophrenia. Bivariate Spearman correlation of (C) apathy (r_s_ = -.01, p = .96) and (D) diminished expression (r_s_ = -.39, p = .07) with percent signal change in the dorsal striatum in healthy controls. BNSS = Brief Negative Symptom Scale.

**Fig 5:**
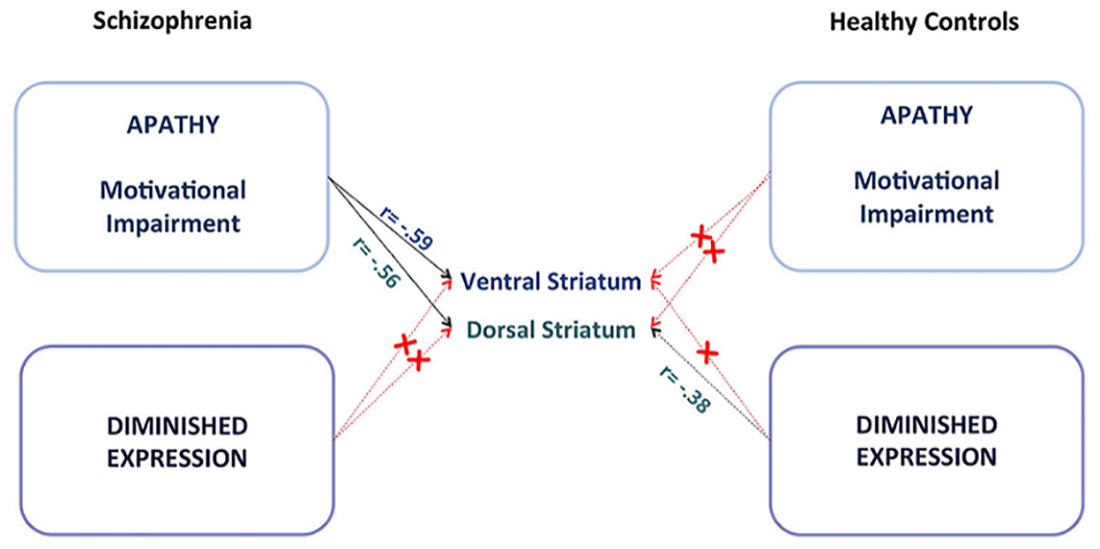
Summary of the main findings. Correlations between the striatal signal during reward anticipation and dimensions of negative symptoms in patients with chronic schizophrenia and healthy participants.

Consistent with our hypothesis, in patients with SZ dorsal striatal activation during reward anticipation correlated negatively with apathy (r_s_ = -.56, p = .02) (Fig 4A). As expected, we found no significant association between DS activation and diminished expression (r_s_ = .17, p = .54) (Fig 4B). Reduced activation of the DS was specifically associated with greater apathy score, as the two correlation coefficients differed significantly from each other (z = -2.1, p = .04). In healthy participants, we found a trend for DS signal during reward anticipation being associated with diminished expression (r_s_ = -.38, p = .07), but not with apathy (r_s_ = -.01, p = .96) (Fig 4C and 4D). However, when combining both factors in the BNSS total score, we found that this measure of global negative symptoms was not associated with DS activity in healthy controls (r_s_ = -.31, p = .17) (Fig C in S1 File). Fig 5 summarized all correlation analyses of both groups in a general overview.

#### No association with confounding variables

Potentially confounding variables in SZ, such as chlorpromazine equivalents (VS: r_s_ = -.05, p = .86; DS: r_s_ = .16, p = .28), positive symptoms (PANSS positive factor: VS: r_s_ = .06, p = .84; DS: r_s_ = .12, p = .67), and depressive symptoms (CDSS: VS: r_s_ = .128, p = .64; DS: r_s_ = .21, p = .22) did not correlate significantly with the percentage signal change during reward anticipation. In HC, depressive symptoms did not correlate with ventral and dorsal striatal activity during reward anticipation (VS: r_s_ = .13 p = .57, DS: r_s_ = -.04, p = .86).

## Discussion

In this study, we replicated our previous finding of a specific association between reduced ventral striatal activation and apathy in an independent sample of patients with chronic schizophrenia [7]. In addition, we observed, for the first time, that apathy but not diminished expression was associated with dorsal striatal activity in schizophrenia patients. These findings add new evidence for striatal dysfunction as a neural substrate underlying apathy. Critically, the present study suggests that both ventral and dorsal striatal activity during reward anticipation contribute to the pathophysiology of motivational impairments in schizophrenia. In healthy individuals with non-clinical levels of negative symptoms we found that diminished expression but not apathy was related to reduced dorsal striatal activity on the trend level. These results suggest that some of the striatal deficits associated with negative symptoms may also be detectable in healthy individuals and underlies the need for further investigation of apathy and diminished expression in the general population.

### Relationship between apathy and ventral striatal dysfunction in chronic schizophrenia

Radua et al’s recent meta- analysis demonstrates that there is consistent evidence for the relationship between reduced VS activation during reward anticipation and the severity of negative symptoms in schizophrenia [18]. Our observation of the specific relationship to apathy in schizophrenia strengthens the existing evidence of the distinct pathophysiological mechanisms that underlie the two negative symptoms dimensions. In accordance with previous research [6,8,23], the present findings support the link of VS dysfunction during reward anticipation with motivational deficits in schizophrenia.

### Relationship between apathy and dorsal striatal dysfunction in chronic schizophrenia

Even though DS activity during reward anticipation has been proposed earlier [54,55], the potential association with negative symptoms has rarely been addressed. Only recently Mucci et al. reported a relationship between reduced dorsal caudate signals during reward anticipation with real-life motivation and avolition, but not with anhedonia [26]. Similar to the clear distinction present in the VS, we observed that DS dysfunction was associated with apathy but not diminished expression. The DS is involved in encoding specific action-outcome associations in goal-directed behavior and the selection of actions on the basis of their currently expected reward value [17]. Critically, it has been suggested that DS activation is more prominent during tasks, in which a participant’s actions directly determines the outcome [56–58]. Therefore, our adaptation of the classic MID paradigm may enable us to observe dysfunction in the DS in patients with SZ. Although it was not the objective of our previous study, a re-analysis of the data revealed no association between reduced DS activity and apathy [6]. One possible explanation for these divergent findings may be differences in the sample characteristics of the two studies on the behavioral level. In contrast to the former study, patients with SZ were significantly slower compared to HC, which was most pronounced in individuals with higher levels of negative symptoms. Furthermore, the present patient group showed slower RTs in all conditions compared to the patient group of our previous study [6]. Although speculative, these effects might be a result of different levels of fatigue during the experimental session. While in the former study participants performed the task at the beginning of the study day, the present sample performed the task after 1 hour of effortful tasks. Hence, one might speculate that in the current study fatigue may have impaired action-outcome coding in the DS and response in patients with high levels of apathy.

### Relationship between apathy/diminished expression and striatal responses in healthy controls

In healthy controls, dorsal, but not ventral striatal activation during reward anticipation was correlated on a trend-level with the severity of diminished expression. These results should be interpreted in the light of very low level of non-clinical NS in the present healthy control sample. So far, the limited literature discussing the association of altered brain activation during reward anticipation with negative symptoms in non-clinical population reports inconclusive findings [8,43]. The use of divergent rating scales and different levels in symptom severity are a plausible explanation for these contradictory findings. Recently an association of apathy with VS hypoactivation across schizophrenia patients and healthy participants with large variance in subclinical symptom expression was reported [43]. Another study probed VS signal to unpredictable reward outcomes during a monetary card guessing task, but did not find an association with negative symptoms in healthy subjects with very low levels of negative symptoms [8]. Taken together, these findings suggest that dysfunctional striatal activation might depend more on the severity of negative symptoms than on the diagnostic status.

It is important to note that due to the lack of validated dimensional assessments it is challenging to measure negative symptoms spanning the clinical and healthy population. Although the BNSS is useful for comparing data between non-clinical and clinical populations it might not be optimal for the assessment of negative symptoms in the general population. Taken together, the association between reduced reward-related signal in the dorsal striatum and low levels of negative symptoms in healthy individuals suggest a dimensional view of symptom expression, which needs further investigation across the general population [43].

### Group comparison of striatal activation during reward anticipation

In the present study neural activation in the ventral and dorsal striatum during reward anticipation did not differ on the group level. Although there is now meta-analytic support for reduced VS activation in patients with schizophrenia, results on the individual study level are inconsistent [6,23,26,59–66]. As proposed previously, one possible explanation for this inconsistency might be the normalizing effect of atypical antipsychotics on striatal activity [6,20,66–68]. An alternative explanation is that patients with low expression of apathy may have intact neural activity in the striatum during reward anticipation, which in turn limits the observation of group differences. Taken together, the divergent findings of group differences on an individual study level suggest that reduced VS activity may be observable only in a subset of schizophrenia patients, with medication and negative symptom levels as potential influencing factors.

### Limitations

Some limitations of this study should be discussed. First, although the presented correlations of striatal hypoactivation with apathy in SZ were strong and highly significant, the relatively small sample size poses a major limitation. Thus, future studies with larger samples and a broad range of non-clinical and clinical symptom levels could facilitate precise targeting of the neural underpinnings of both negative symptom domains. Second, patients were medicated with atypical antipsychotics. Despite the fact, that the chlorpromazine equivalent dose did not have any statistical effect, our findings may not be generalizable to un-medicated patients or to those receiving typical antipsychotics. Third, although the MID task consistently shows a robust signal during reward anticipation and participants were trained on the cue-outcome contingencies before performing the task, the influence of prediction error signals on the subsequent anticipation phase cannot be fully excluded. However, in contrast to previous reward anticipation studies [24,63,68–71], we avoid win/loss probability and reward/no-reward dichotomies in our task design to minimize the effect of prediction error signals during the outcome phase on the subsequent reward anticipation phase. Fourth, as we did not use the expression of NS as inclusion criterion for our healthy controls sample, the average value for the two negative symptom factors were very low, what is the most probable reason for our negative results in the HC group. Finally, we did not directly measure fatigue, which may at least partly account for our behavioral and neural findings. Future studies, particularly those consisting of long or effortful tasks should include the measurement of fatigue as an important variable, so that we can interpret its impact on behavioral results and neural correlates of negative symptoms.

### Conclusion

The specific correlation of reduced ventral and dorsal striatal signal and apathy in chronic schizophrenia patients strengthens the existing evidence for distinct pathophysiological mechanisms underlying the two negative symptom domains. Our findings suggest that not only ventral but also dorsal striatal dysfunction underlies the complex construct of motivational deficits in schizophrenia.

## Supporting information

S1 File Supplemental information. (DOCX)
