# Supplementary material for: Investigating the association of ventral and dorsal striatal dysfunction during reward anticipation with negative symptoms in patients with schizophrenia and healthy individuals
Source: PLoS One. 2018 Jun 18;13(6):e0198215. doi: 10.1371/journal.pone.0198215 (PMC6005482; doi:10.1371/journal.pone.0198215)
Supplement: S1 File — (DOCX) [file pone.0198215.s001.docx]

**Investigating the association of ventral and dorsal striatal dysfunction during reward anticipation with negative symptoms in patients with schizophrenia and healthy individuals.**

Supplemental Information

1. **S1 Fig A. Graphical illustration of pay-out structure.**

Pay-out structure of the Monetary Incentive Delay task variant Adapted from Simon et al (1). For every individual, we calculated the 15 previous response times and sorted them from fast to slow using a simple bubble sorting procedure. We then selected the response times corresponding to the 60^th^ percentile and 80^th^ percentile, defining a minimum and maximum of the time range within each participant had to respond in order to win money (grey area = time range 0 to 1). Due to the fact that these ranges tended to be small (~5 to 10 milliseconds), we dispersed the time frame, ranging from -2.5 ranges below the original minimum and 1 range above the original maximum. Finally, the pay-out amount was determined by plotting the amount won in each trial with this modified dispersion of the original time range. With respect to the pay-out structure this approach gave the task a realistic feel by providing more dispersed outcome amounts. The X-axis represents the corresponding percentage of the maximal possible win during each trial. For example: in order to win 40% of the maximum amount during the CHF 2 condition (40%=CHF 0.8), participants had to respond at -1 range below the minimum. In order to win 80% (CHF 1.6), the response time had to be 1 range above the minimum.

1. **S1 Table A. Group comparison of payment during the fMRI task**

| Payment |  |  |  |  |  |  |  |  |  |
| --- | --- | --- | --- | --- | --- | --- | --- | --- | --- |
|  |  | N | Min. | Max. | Mean | SD | t-test | df | p |
| mean_payment_low | HC | 23 | 0.19 | 0.33 | 0.27 | 0.04 | 0.15 | 37 | 0.89 |
|  | SZ | 16 | 0.19 | 0.31 | 0.27 | 0.03 |  |  |  |
| mean_payment_high | HC | 23 | 1.24 | 1.77 | 1.52 | 0.14 | 3.44 | 37 | 0.0010 |
|  | SZ | 16 | 1.14 | 1.53 | 1.39 | 0.1 |  |  |  |
| mean_payment | HC | 23 | 0.47 | 0.7 | 0.6 | 0.06 | 2.11 | 37 | 0.04 |
|  | SZ | 16 | 0.47 | 0.65 | 0.56 | 0.05 |  |  |  |
| total_payment | HC | 23 | 31.3 | 47.99 | 40.87 | 4.42 | 3.13 | 37 | 0.003 |
|  | SZ | 16 | 27.31 | 41.26 | 36.67 | 3.64 |  |  |  |

1. **S1 Table B. Voxel-wise whole brain analysis of the contrast high reward anticipation vs. no reward anticipation, across all subjects, FWE-voxel-level-corrected p<0.05.**

| Hem. |  | BA | MNI coordinates (x, y, z) | T | cluster size | p(FWE) |
| --- | --- | --- | --- | --- | --- | --- |
| R | Insula | 13 | 35 18 3 | 9.09 | 524 | <0.001 |
| R | Insula | 13 | 35 24 -5 | 7.5 |  |  |
| R | Caudate Head |  | 8 15 -5 | 8.65 | 215 | <0.001 |
| R | Anterior Cingulate | 24 | 0 21 18 | 8.62 | 554 | <0.001 |
| R | Anterior Cingulate | 32 | 8 30 22 | 7.62 |  |  |
| R | Cingulate Gyrus | 24 | 3 9 28 | 7.06 |  |  |
| R | Culmen |  | 29 -52 -27 | 8.59 | 283 | <0.001 |
| L | Caudate Head |  | -8 17 -3 | 8.19 | 149 | <0.001 |
| L | Medial Globus Pallidus |  | -12 0 -6 | 8.15 | 153 | <0.001 |
| L | Putamen |  | -15 6 -12 | 7.89 |  |  |
| L | Precentral Gyrus | 4 | -35 -25 54 | 8.03 | 534 | <0.001 |
| L | Postcentral Gyrus | 3 | -41 -21 51 | 7.57 |  |  |
| R | Postcentral Gyrus | 4 | -39 -30 48 | 6.23 |  |  |
| R | Superior Frontal Gyrus | 6 | 2 9 46 | 8.01 | 215 | <0.001 |
| R | Cingulate Gyrus | 32 | 9 11 40 | 6.42 |  |  |
| R | Thalamus |  | 6 -22 -0 | 7.42 | 59 | <0.001 |
| R | Middle Frontal Gyrus | 6 | 30 -3 45 | 7.41 | 223 | <0.001 |
| R | Middle Frontal Gyrus | 6 | 23 3 46 | 6.87 |  |  |
| L | Midbrain, Red Nucleus |  | -5 -25 -5 | 7.38 | 60 | <0.001 |
| L | Midbrain |  | -3 -18 -17 | 7.32 | 42 | <0.001 |
| L | Middle Frontal Gyrus | 6 | -23 -4 48 | 6.98 | 96 | <0.001 |
| L | Cingulate Gyrus | 24 | -3 -1 49 | 6.98 | 38 | <0.001 |
| R | Medial Globus Pallidus |  | 14 -3 -6 | 6.97 | 17 | 0.002 |
| R | Putamen |  | 15 6 -12 | 6.89 | 30 | <0.001 |
| L | Midbrain, Red Nucleus |  | -6 -16 -9 | 6.87 | 27 | <0.001 |
| L | Middle Frontal Gyrus | 6 | -35 -3 46 | 6.65 | 14 | 0.003 |
| R | Cerebellum, Inf. Semi-Lunar Lobule |  | 6 -70 -39 | 6.63 | 20 | 0.001 |
| R | Middle Frontal Gyrus | 17 | 11 -91 3 | 6.52 | 20 | 0.001 |
| R | Caudate Body |  | 12 0 16 | 6.52 | 20 | 0.001 |
| L | Thalamus, Medial Dorsal Nucleus |  | -5 -18 13 | 6.51 | 35 | <0.001 |
| R | Thalamus, Medial Dorsal Nucleus |  | 5 -15 13 | 6.15 |  |  |
| L | Postcentral Gyrus | 2 | -45 -30 36 | 6.5 | 57 | <0.001 |
| L | Inferior Parietal Lobule | 4 | -36 -37 36 | 6.14 |  |  |
| R | Midbrain, Substantia Nigra |  | 12 -12 -11 | 6.49 | 21 | 0.001 |
| R | Inferior Frontal Gyrus | 9 | 45 9 27 | 6.46 | 32 | <0.001 |
| R | Inferior Frontal Gyrus | 9 | 35 11 24 | 6.38 |  |  |
| R | Cereberral Vermis, Declive |  | 3 -69 -18 | 6.35 | 26 | 0.001 |

Notes: Hem. = Hemisphere; BA = Brodman Area

1. **S1 Fig B. Correlations of the global negative symptoms with VS activation during reward anticipation.**


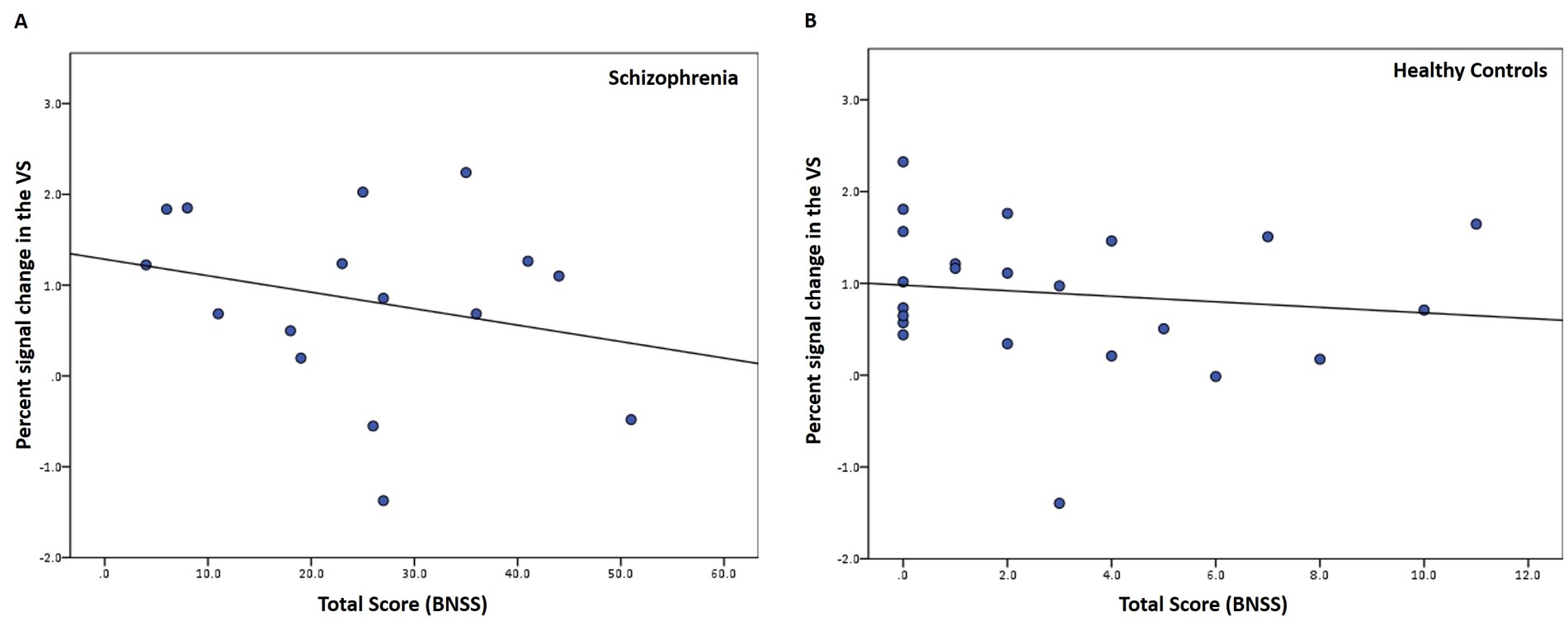


Bivariate Spearman correlation of global negative symptoms with percent signal change in the ventral striatum in (A) patients with schizophrenia (r_s_=-.21, p=.44), (B) healthy controls (r_s_=-.24, p=.27),. BNSS = Brief Negative Symptom Scale.

1. **S1 Fig C. Correlations of the global negative symptoms with DS activation during reward anticipation.**


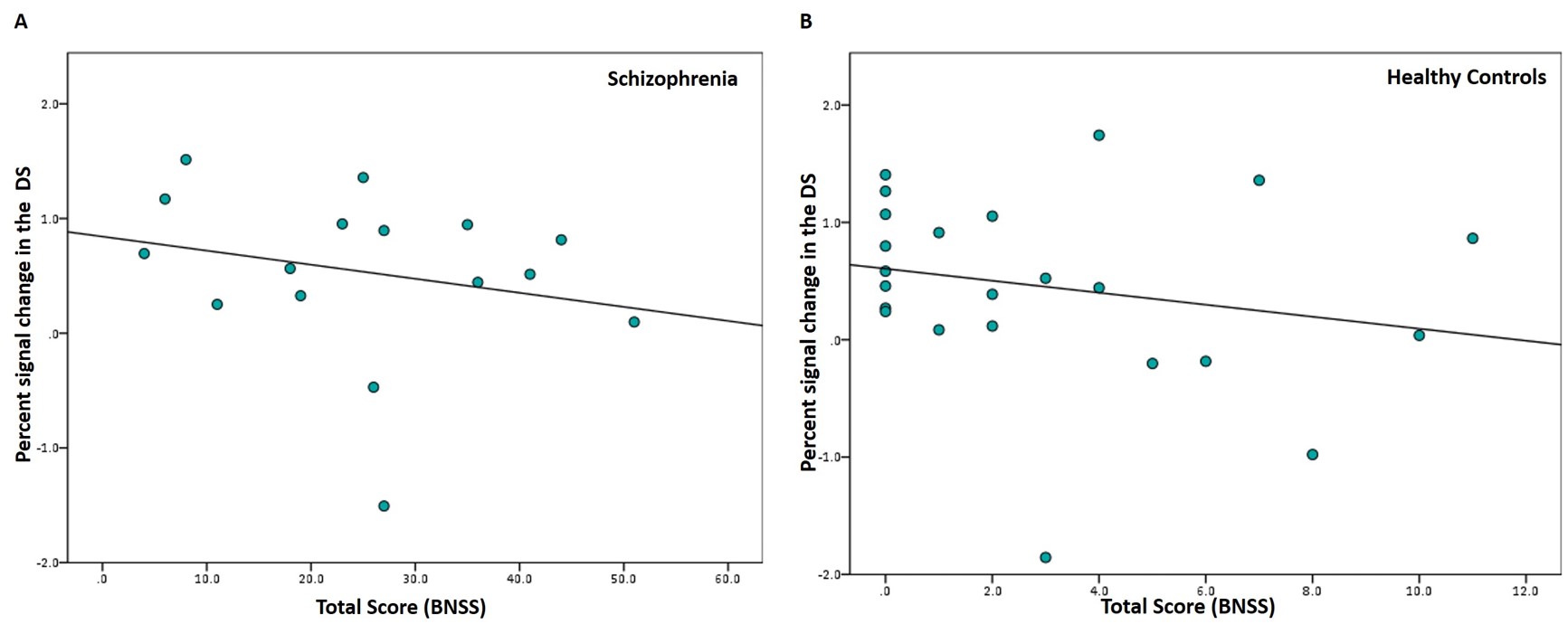


Bivariate Spearman correlation of global negative symptoms with percent signal change in the dorsal striatum in (A) patients with schizophrenia (r_s_=-.33, p=.21), (B) healthy controls (r_s_=-.31, p=.17). BNSS = Brief Negative Symptom Scale.

1. **References**

1. Simon JJ, Cordeiro SA, Weber M-A, Friederich H-C, Wolf RC, Weisbrod M, Kaiser S (2015): Reward System Dysfunction as a Neural Substrate of Symptom Expression Across the General Population and Patients With Schizophrenia. *Schizophr Bull*. 41: 1370–1378.
